# Supplementary material for: Acceleration of Electrospun PLA Degradation by Addition of Gelatin
Source: Int J Mol Sci. 2023 Feb 10;24(4):3535. doi: 10.3390/ijms24043535 (PMC9966984; doi:10.3390/ijms24043535)
Supplement: Supplementary file 1 [file ijms-24-03535-s001.zip › ijms-2149678-supplementary.pdf]

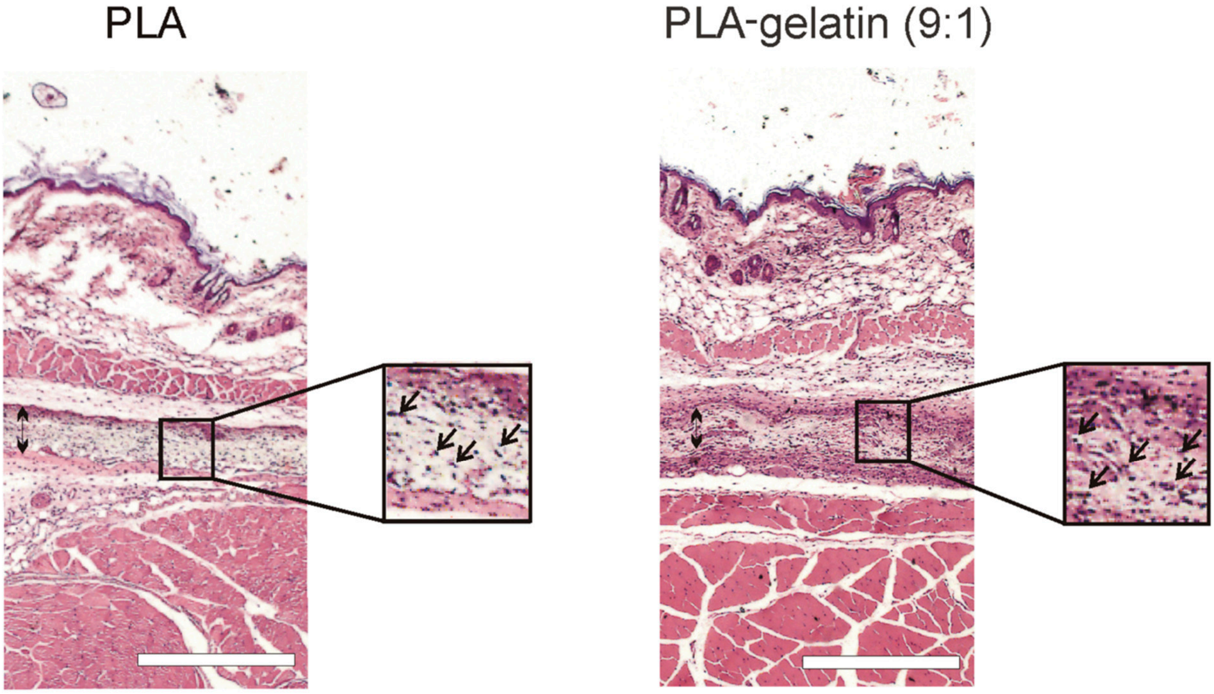

Supplementary Figure S1. Infiltration of PLA and PLA-gelatin electrospun mats with host cells on day 14 after subcutaneous implantation to C57BL/6 mice. Double-ended arrow indicates the thickness of the implanted biomaterial. Insets of the main figures represent the areas of the implanted biomaterials infiltrated with the host cells (black arrows). Scale bar is 500 μm.
